# Compositionally Controlled Electron Transfer in Metallo-Organics

**DOI:** 10.1021/jacs.3c05874

**Published:** 2023-08-02

**Authors:** Yonatan Hamo, Alena Neudert, Tatyana Bendikov, Michal Lahav, Milko E. van der Boom

**Affiliations:** † Department of Molecular Chemistry and Materials Science, The Weizmann Institute of Science, 7610001 Rehovot, Israel; ‡ Department of Chemical Research Support, The Weizmann Institute of Science, 7610001 Rehovot, Israel

## Abstract

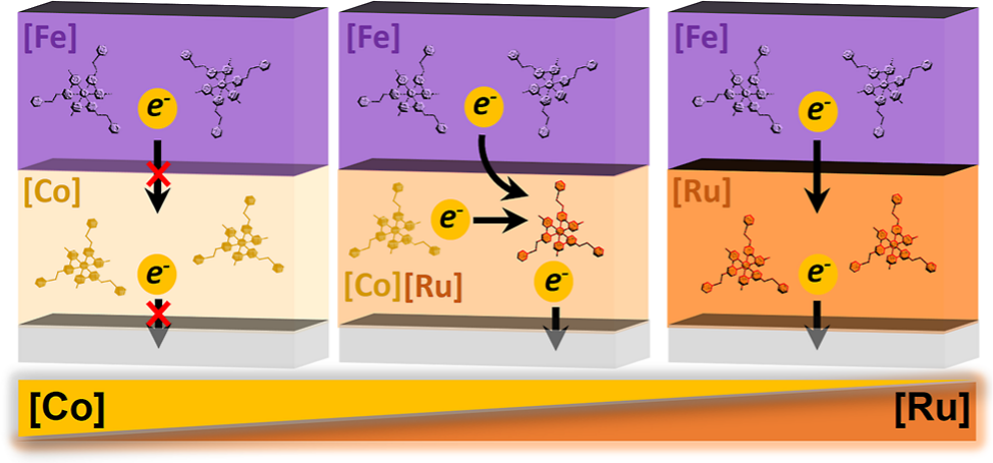

We demonstrate here the assembly of a nanolayer of electrochromic iron complexes on the top of composite layers of cobalt and ruthenium complexes. Depending on the ratio of the latter two complexes, we can tailor materials that show different electron transport pathways, redox activities, and color transitions. No redox activity of the top layer, consisting of iron complexes, is observable when the relative amount of the ruthenium complexes is low in the underlying composite layer because of the insulating properties of the isostructural cobalt complexes. Increasing the amount of ruthenium complexes opens an electron transport channel, resulting in charge storage in both the cobalt and iron complexes. The trapped charges can be chemically released by redox-active ferrocyanide complexes at the film–water interface.

## Introduction

The fundamental surface chemistry of relatively simple hydrocarbons is considered as one of the sources from which the fields of nanoscience and technology emerged. The seminal works about the self-assembly and development of the monolayers of Sagiv, Nuzzo, Allara, Whitesides, and many others form the basis of many functional interfaces and materials.^1−6^ Increasingly, complex and functional organic molecules have been used by many others to introduce surface-bound molecular machinery, optical properties, and binding sites for biomolecules, for example.^7−15^ The binding of molecules from the gas phase or solution onto a solid support is a frequently used approach to form nanostructures and thin films.^16−21^ There is an excellent compatibility of many organic functional groups with diverse substrate surfaces (e.g., alkyl thiols and silanes with metallic and hydrophilic surfaces, respectively).^22,23^ Most thin films consist of only one molecular component. Despite many studies, control over the formation of multicomponent materials remains challenging. Different chemical and structural properties of the molecular components can cause self-sorting, phase separation, and aggregation, resulting in the formation of domains.^24,25^ The molecular composition of feed solutions is not always reflected in the thin film structure; similar observations were made regarding the growth of co-crystals from solutions containing more than one organic component.^26−28^ Control over the composition of supramolecular thin films is intriguing since new electronic properties and other useful functionalities can arise.^29−34^

The use of coordination chemistry for the formation of redox-active assemblies on conductive surfaces resulted in properties that are not readily possible with other materials. The long-range electron transfer characteristics of such assemblies were attributed to structural (e.g., porosity) and electronic factors. Electron hopping and more recently a “stepping-stone mechanism” (=modified tunneling process) were proposed.^35,36^ Assemblies having reversible electron transfer to unidirectional current flows and even charge trapping have been demonstrated.^37^ Reversible changes in the metal oxidation state, along with concurrent large variations in their light absorption efficiency, have resulted in potentially useful electrochromic coatings.^38,39^ Electrocatalysis, sensing, and antibacterial properties have been reported as well as supercapacitors, electrochromic devices, and inverted solar cells.^39−49^ Such films have also been shown to mimic the characteristics of conventional electronic circuits, including flip-flops.^50,51^ The functionalities of metal–organic films can be controlled by sequence-dependent assembly, that is, depositing nanoscale layers consisting of isostructural complexes with different metal cations.^19,52,53^

Molecular assemblies (MAs) based on ruthenium polypyridyl complexes have been demonstrated as efficient electron-transporting layers for inverted organic photovoltaic cells.^54^ Key to the performance of these cells is the electron-transporting material that functions as an electron transport/hole blocking layer. In dye-sensitized solar cells, a current flow is generated by injecting electrons into TiO_2_ electrodes from ruthenium polypyridyl complexes, which are subsequently being reduced by the iodine electrolyte. More recently, cobalt complexes have replaced iodine as the redox mediator.^55^ Unidirectional current flow is also of great importance in electronic components such as Zener diodes. Organic thin films can exhibit similar properties, but the design of such systems is not straightforward as most conductive systems display bidirectional electron transfer and not versatile.^37,56^

**Scheme 1:**
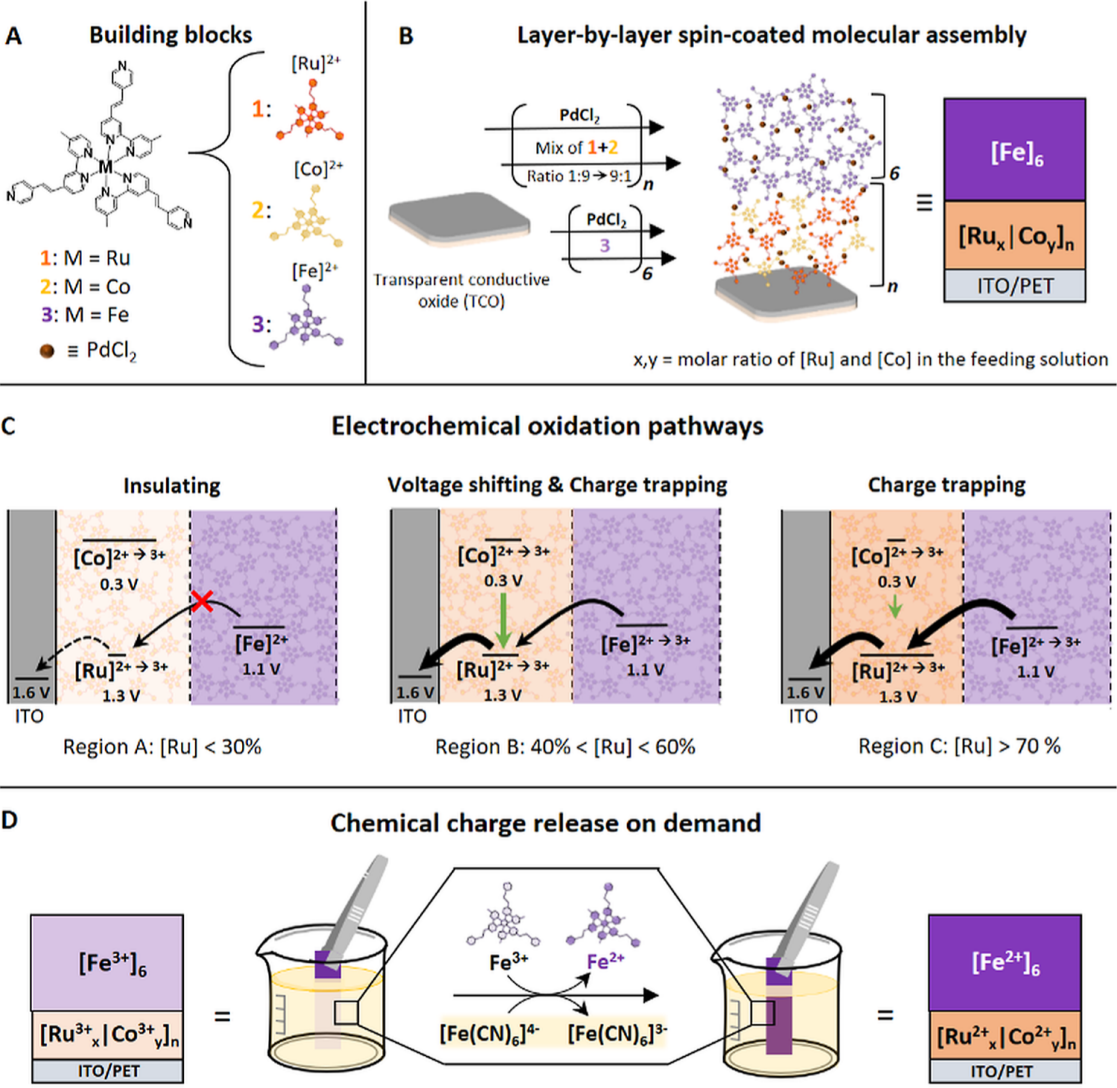
Metallo-Organic Assemblies (MAs) and Their Composition, Electrochemical Properties, and Chemical Charge Release (A) The molecular components. The counterions (PF_6_^–^) have been omitted for clarity. (B) Film formation: solutions containing different percentages of ruthenium (**1**) and cobalt (**2**) complexes, as well as solutions of PdCl_2_(PhCN)_2_, were deposited on ITO/PET by alternatively layer-by-layer spin-coating. The iron complex (**3**) was deposited using identical conditions. Surface assembly sequence: *n* = 3–4 → the number of deposition cycles. (C) Correlation between the charge transport and the ratio of complexes **1** and **2** in [Ru_*x*_|Co_*y*_]_*n*_[Fe]_6_. (D) Chemical charge release.

In this study, we combine such ruthenium–cobalt redox couple in thin films (Scheme 1). By using a combination of isostructural metal complexes, we obtained a series of materials that display a full range of electrochemical properties: insulation, reversible redox chemistry, unidirectional electron transfer, and charge trapping. These properties that cannot be achieved otherwise are a result of redox communication between the ruthenium (**1**) and cobalt (**2**) complexes. The latter component is electrochemically silent on metal oxide surfaces but can undergo electron transfer with the ruthenium complexes: the cobalt (**2**) complexes are oxidized at the onset oxidation potential of the ruthenium (**1**) complexes. Complexes **1** mediate the electron transfer to the metal oxide surface. Since this process occurs under an oxidative potential, the ruthenium (**1**) complexes are being oxidized. Changing the ratios between these homogeneously distributed complexes in the film gave rise to a diverse electrochemical behavior. An electrochromic layer of iron (**3**) complexes on the top was used here as an indicator for the charge transfer properties of the series of mixtures of the ruthenium (**1**) and cobalt (**2**) redox couples. Electrochemically trapped charges can be released chemically by an electron donor in solution. Mechanistically, this electron transfer takes place at the solution–surface interface. Although the external electron donor is only in direct contact with the film surface, the underlying metal complexes undergo fast self-reduction, and the charge storage state is reflected in the coloration of our assemblies.

The coordination chemistry from solution to the surface to form these mixed metal complex films is not straightforward. Extensive characterization showed that the films consisting of mixtures of metal complexes do not reflect their molecular ratios in the feed solution.

## Results and Discussion

Solutions containing different ratios of complexes **1** and **2** were spin-coated on indium tin oxide (ITO)/polyethylene terephthalate (PET) (3 cm × 3 cm), forming [Ru_*x*_|Co_*y*_]_*n*_. Note: *x* and *y* represent their molar ratios in the feeding solutions, respectively, and *n* is the number of depositing cycles (Scheme 1B). Subsequently, a layer of complex **3** was deposited to afford [Ru_*x*_|Co_*y*_]_*n*_[Fe]_6_. Palladium dichloride served as a linker for attaching the complexes to the substrate surfaces as well as a linker for the MAs by coordination with the vinylpyridine moieties.^53^ The deposition sequence for the formation of [Ru_*x*_|Co_*y*_]_*n*_ was repeated three to four times in order to reach a thickness that prohibits direct electrochemical communication between the conductive support and the top layer consisting of complex **3** (Figure S1).

**Figure 1:**
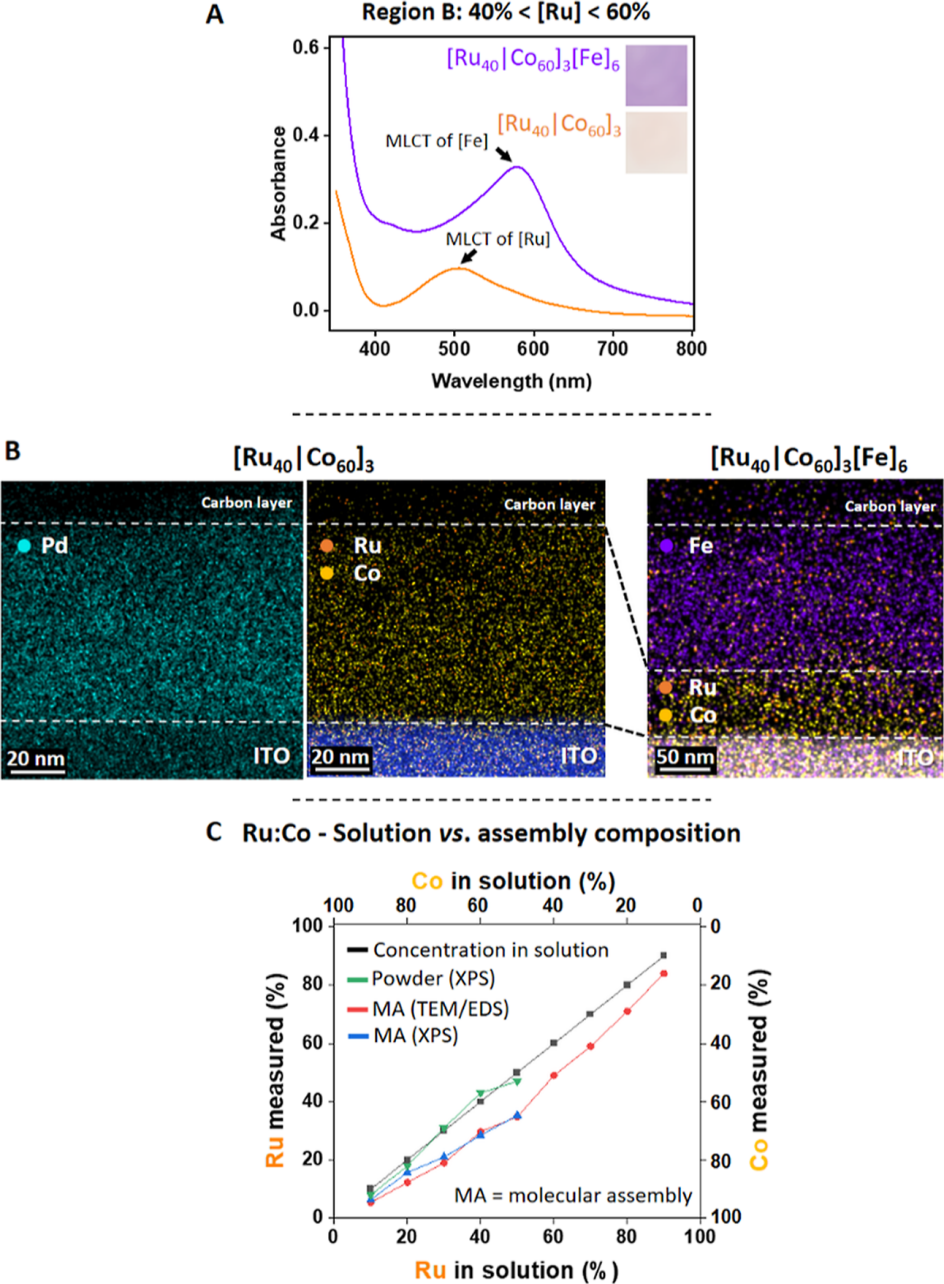
Formation and characterization of the MAs on ITO/PET. (A) UV/vis spectra of [Ru_40_|Co_60_]_3_, orange, and [Ru_40_|Co_60_]_3_[Fe]_6_, purple (region B). Inset: Photographs of the modified surfaces (1.5 cm × 1.5 cm). (B) TEM–EDS images of [Ru_40_|Co_60_]_3_ and [Ru_40_|Co_60_]_3_[Fe]_6_. (C) Chemical composition of [Ru_40_|Co_60_]_3_, measured by TEM–EDS (red) and XPS (blue) for the MAs as well as the dried solution mixtures of **1** and **2** (green) without PdCl_2_, as measured by XPS. The graph also shows the anticipated ratios in solution (black).

The films, [Ru_*x*_|Co_*y*_]_*n*_ and [Ru_*x*_|Co_*y*_]_*n*_[Fe]_6_, were characterized by UV/vis spectroscopy (Figure S3). A representative UV/vis spectrum of [Ru_40_|Co_60_]_3_ shows the expected metal-to-ligand charge transfer (MLCT) bands at λ_max_ = 500 nm, corresponding to complex **1** (Figure 1A, orange). Cobalt complex **2** absorbs only weakly in the visible region; therefore, its contribution to the color of the films is nihil (Figure S4). The corresponding spectrum of [Ru_40_|Co_60_]_3_[Fe]_6_ (Figure 1A, purple) was measured after six deposition steps of the iron complex **3** on the top of [Ru_40_|Co_60_]_3_. A broad band at λ_max_ = 570 nm was observed for the MLCT band of complex **3**, similar to the UV/vis spectrum of the complex in solution (Figure S4).^7^ A linear film growth is observed (Figure S3B, inset). The films were further analyzed using transmission electron microscopy (TEM), combined with energy-dispersive X-ray spectroscopy (EDS), to estimate the thickness and to determine the elemental composition and the distribution of the metal complexes. Representative examples of TEM–EDS measurements of [Ru_40_|Co_60_]_3_ and [Ru_40_|Co_60_]_3_[Fe]_6_ are shown in Figure 1B. The samples were coated with a thin layer of carbon and platinum to avoid damage to the organic layer and milled using a focused ion beam microscope.

Analyses of the resulting cross-sections showed that the films are continuous: no cracks or defects are visible. The estimated thicknesses decrease from ∼83 nm for [Ru_10_|Co_90_]_3_ to ∼67 nm for [Ru_60_|Co_40_]_3_. Therefore, a fourth deposition cycle was added for these assemblies using solutions containing 70–90% of complex 1. This additional layer resulted in assemblies with thicknesses of ∼92 nm for [Ru_70_|Co_30_]_4_ to ∼71 nm for [Ru_90_|Co_10_]_4_. The thickness of [Fe]_6_ within [Ru_40_|Co_60_]_3_[Fe]_6_ is ∼133 nm. We observed an even distribution of various elements (Ru, Co, Fe, Pd, N, and Cl), indicating the formation of homogeneous structures. There is no evidence of physical clustering of complexes **1**–**3**. A lower amount of complex **1** was observed on the surface than anticipated (∼30% instead of 40%); concomitantly, the amount of complex **2** was higher than expected by ∼10%. For all [Ru_*x*_|Co_*y*_]_*n*_ layers, the expected ratios between the metal complexes on the surfaces differ from the ratios used in the feed solutions by 8.6 ± 2.4% (the black and red graphs in Figure 1C). This effect was confirmed by further analysis of the composition of [Ru_*x*_|Co_*y*_]_*n*_ using X-ray photoelectron spectroscopy (XPS) for five samples prepared by depositing solutions with increasing percentages of complex **1** from 10 to 50% in steps of 10%. Although XPS is a surface analytical method,^57^ here, the structure of the MAs allowed us to reach a depth of 10–15 nm. The same trend as that observed by TEM–EDS was also observed in XPS (the blue graph in Figure 1C). The data revealed equally lower and higher percentages of complexes **1** and **2**, respectively, compared to the expected ratios. To verify that the feed solutions contained both the metal complexes in the expected ratios and to rigorously exclude any experimental errors, we also analyzed drop-casted samples on ITO by XPS. These measurements matched well with the anticipated ratios of complexes **1** and **2** (the green graph in Figure 1C). The experimentally observed ratios between the two isostructural complexes might reflect the relative rates of the binding of the complexes to the surface with *k*_2_ > *k*_1_. The origin of the higher reactivity of the cobalt complex (**2**) involves various factors (facial and meridional isomers, packing, and electronic properties). We have shown previously that these and other polypyridyl complexes can undergo sorting from the solution to surface.^58^

We wish to better understand the influence of different ratios of the ruthenium (**1**) and cobalt (**2**) complexes on the redox chemistry of the top layer consisting of iron complexes (**3**). Therefore, the compositions of the feeding solutions were varied from 0 to 100% for complex **1** (in steps of 10%), the rest being complex **2**. We first analyzed the effect of the electrochemical properties of [Ru_*x*_|Co_*y*_]_*n*_ on ITO using cyclic voltammetry (CV), with a potential window of 0.2–1.8 V vs Ag/Ag^+^. Based on their electrochemical behavior, the results were divided into three distinct regions: region A = 0–30%, region B = 40–60%, and region C = 70–100% of complex **1** in the feeding solutions (Scheme 1C).

**Figure 2:**
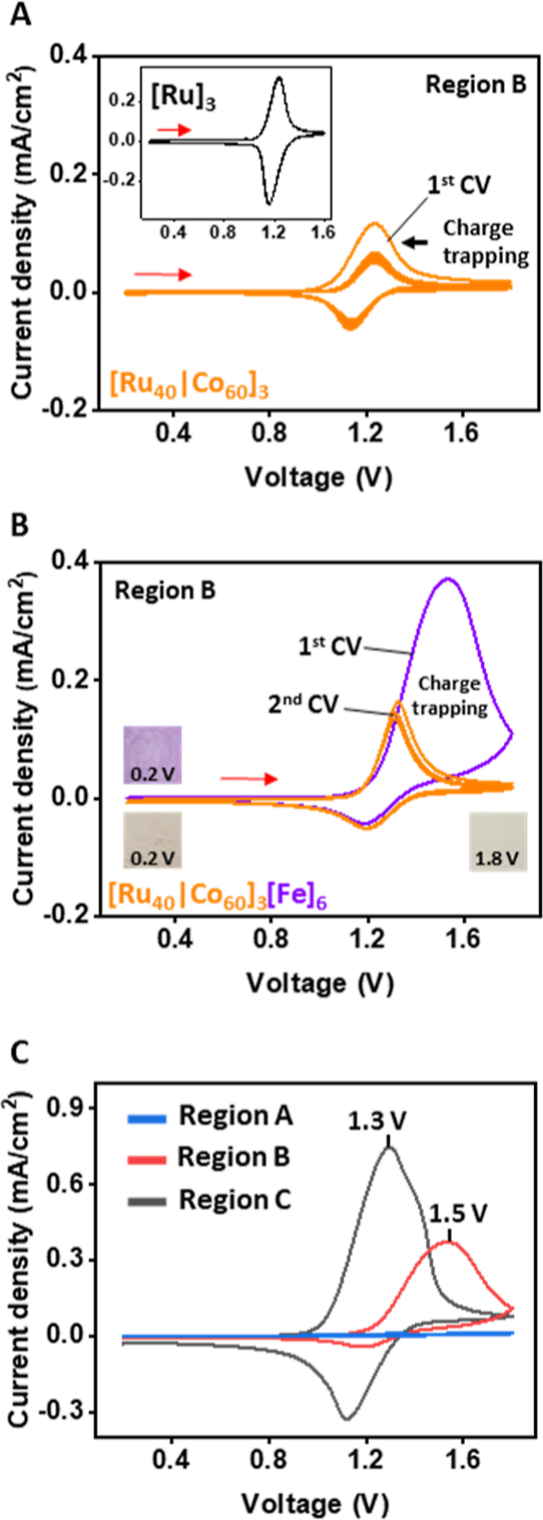
CV measurements of (A) [Ru_40_|Co_60_]_3_. Inset: two CV curves of [Ru]_3_. (B) [Ru_40_|Co_60_]_3_[Fe]_6_. Insets: photographs showing the modified surfaces before and after oxidation and after a full CV cycle. The red arrow shows the scan direction. (C) 1st cycle of the CV curves of [Ru_20_|Co_80_]_3_[Fe]_6_ (region A), [Ru_40_|Co_60_]_3_[Fe]_6_ (region B), and [Ru_90_|Co_10_]_4_[Fe]_6_ (region C). The measurements were performed in a 0.1 M ACN solution of TBAPF_6_ with ITO/PET, Pt wire, and Ag/Ag^+^ wire as the working, counter, and reference electrodes, respectively.

Here, we focused on [Ru_40_|Co_60_]_3_ as a representative example of region B (Figure 2). The first CV scan of [Ru_40_|Co_60_]_3_ shows an oxidation peak at *E*_ox_ = 1.25 V with *Q*_1st cycle_ = 0.26 mC cm^–2^, which can be associated with the oxidation of both complexes **1** and **2** (Figure 2A). The reduction peak appeared at *E*_red_ = 1.15 V and represents the reduction of complex **1** (Ru^3+/2+^). The second scan shows a clear decrease in the oxidation current (*Q*_2nd cycle_ = 0.11 mC cm^–2^ and Δ*Q* = 0.15 mC cm^–2^). This Δ*Q* indicates charge trapping by cobalt complex **2** after the first cycle; consecutive CV scans (2–5) show the redox behavior of complex **1**. The amount of charge involved in the oxidation processes during the first CV scan corresponds to one-electron oxidation of both ruthenium (**1**) and cobalt (**2**) complexes (M^2+/3+^). Interestingly, the amount of charge involved for the consecutive cycles correlates well with the oxidation of only complex **1**. In support of these assumptions, the ratio between *Q*_2nd cycle_ (=Ru^2+/3+^) and Δ*Q* (=Co^3+^, charge trapped) = 1:1.4 matches with the experimental ratio between complexes **1** (42%) and **2** (58%) = 1:1.4. A decrease of charges is not seen when measuring the CV curves of films consisting of only ruthenium complex **1**: [Ru]_3_ (Figure 2A inset). The differences between the first and second CV cycles with respect to the detected current, shape, and redox potential are negligible. This electrochemical experiment shows that the differences between the first and second CV curves in [Ru_40_|Co_60_]_3_ are due to the involvement of ruthenium complex **1** in the redox process of cobalt complex **2**. The UV/vis measurements of [Ru_40_|Co_60_]_3_ are in agreement with the charge trapping by Co^2+^ (Figure S5A). The absorption band at λ = 500 nm, representing ruthenium complex **1**, disappears upon oxidation (light-orange to transparent); it regains its initial intensity when the film is reduced. Concurrently, the intensity of the π–π* transition peak at λ = 370 nm increases upon oxidation. After a full redox cycle, the intensity of this absorption band does not regain its initial intensity, indicating that the cobalt complexes (**2**) remained in their oxidized state [Co]^3+^. The CV curve of [Ru_40_|Co_60_]_3_[Fe]_6_, as a representative of region B, shows a significant anodic peak (*Q*_1st cycle_ = 0.93 mC cm^–2^), and the purple film becomes transparent (Figure 2B, purple curve and photographs 0.2 V → 1.8 V). The spectroelectrochemical data of [Ru_40_|Co_60_]_3_[Fe]_6_ shown in Figure S5B are in agreement with the abovementioned electrochemical observations and indicate the oxidation of the three complexes and charge trapping in the cobalt (**2**) and iron (**3**) complexes. At 1.6 V, the broad MLCT bands of the ruthenium (**1**) and iron (**3**) complexes disappeared, indicating the successful oxidation of the metal cations (M^2+/3+^). Concurrently, an intense band appeared at λ = 375 nm. Upon applying a reductive potential (0.2 V), the MLCT band of the Ru^3+^ complexes reappeared at λ = 500 nm. A smaller band at λ = 570 nm is also observable, showing that some of the Fe^2+^ species are also oxidized. The new band at λ = 375 nm diminished somewhat in intensity but clearly remained, indicating the irreversible oxidation of the cobalt (**2**) complexes (Figure S5B, inset). These observations are in line withthe electrochemical and spectroelectrochemical behavior of [Ru_40_|Co_60_]_3_ shown above (Figures 2A and S5A). Since no direct communication between the top layer of [Fe]_6_ and the ITO surface can occur, the large amount of ruthenium complexes (**1**) facilitates here the electron transfer from both the cobalt (**2**) and iron (**3**) complexes to the working electrode (Scheme 1C, region B). The oxidative charge, calculated from the area under the curve (Figure 2B, purple curve), in the first CV cycle, is 4.7 times larger than the oxidative charge observed for the second cycle. The concurrent changes in the optical properties of the films indicate the storage of a positive charge in the top layer consisting of iron complexes (**3**) as well as the cobalt complexes (**2**). During the reduction of [Ru_40_|Co_60_]_3_[Fe]_6_, when scanning from 1.8 to 0.2 V, only a single cathodic peak appeared at 1.19 V, and the coloration of the film changed as a result of the reduction of complex **1** (Ru^3+/2+^). Continued cycling shows an anodic peak at 1.30 V (*Q*_2nd cycle_ = 0.25 mC cm^–2^; orange curve) followed by a cathodic peak at 1.11 V that is smaller by ∼24% (*Q*_2nd cycle_ = 0.19 mC cm^–2^). These anodic and cathodic charges become nearly equal upon increasing the scan rate from 100 to 300 mV/s. This effect is typical for an electrochemical–chemical (ECC) process. Such a process does not occur for [Ru_40_|Co_60_]_3_ (Figure 2A). Therefore, we believe that the ECC process for [Ru_40_|Co_60_]_3_[Fe]_6_ is associated with complex **3**. Further support for the occurrence of a chemical reduction pathway is indicated by applying open-circuit potential for the oxidized film (1.8 V, 10 s) and measuring the absorbance by UV/vis spectroscopy. The trapped charges are released in ∼20 min, and the film becomes purple.

The use of [Ru_40_|Co_60_]_3_ results in unidirectional charge transport and trapping in the top layer of [Fe]_6_. The effect is also evident by spectroelectrochemical measurements (Figure S6). The following conditions were applied: (i) 1.6 V_ox_, 10 s, (ii) 0.2 V_red_, 10 s, and (iii) open-circuit potential. The absorbance was measured at λ = 570 nm. About 10% of the charge was released when applying 0.2 V_red_ (Ru^3+→2+^). The iron complexes remained in their higher oxidation state (Fe^3+^). In contrast, applying the same reaction conditions to a film consisting of only [Fe]_6_ (in the oxidized state, Fe^3+^) resulted in electrochemical reduction of the iron complexes (Fe^3+→2+^). As expected, no charge trapping occurred in [Fe]_6_ because of the absence of [Ru_40_|Co_60_]_3_, and the film turned purple.

**Figure 3:**
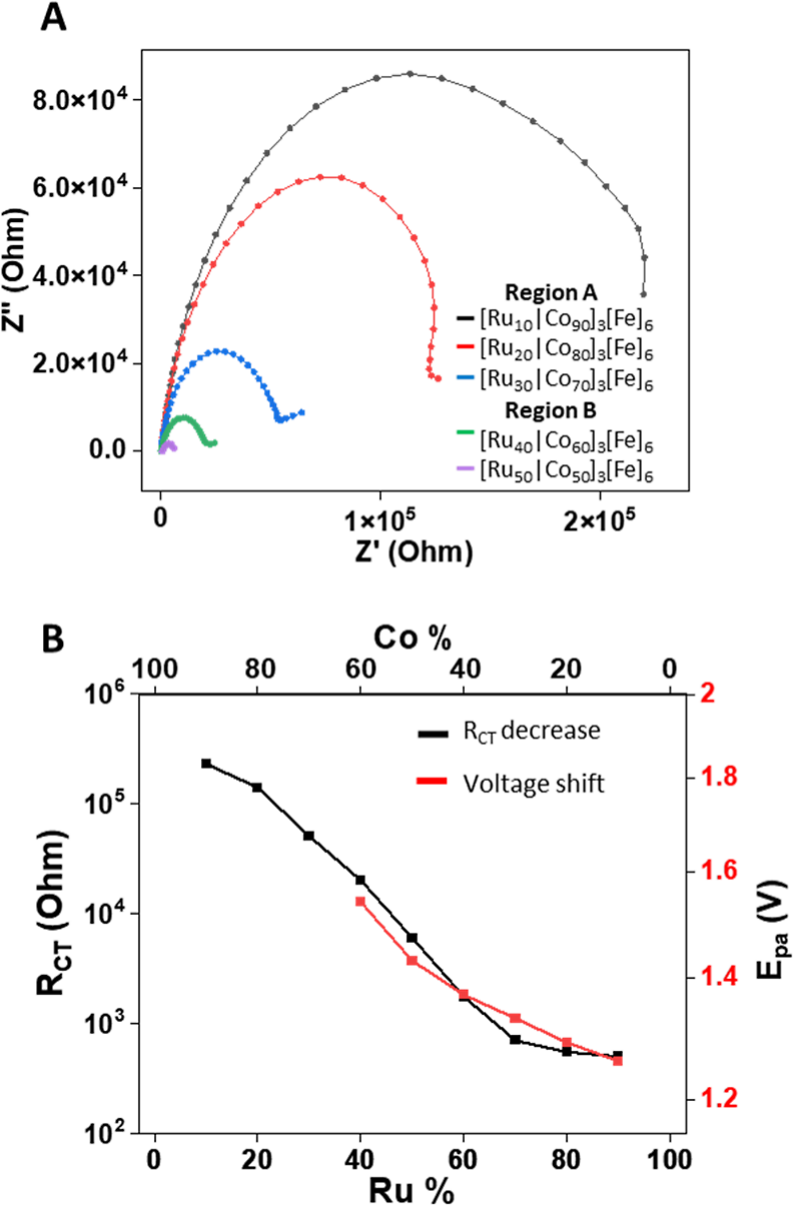
(A) Nyquist plot of [Ru_*x*_|Co_*y*_]_3_[Fe]_6_ derived from the EIS: frequencies of 0.1–10^6^ Hz, amplitude: 10 mV, and *E*^0^ = 1.2 V. (B) Correlation between the *R*_ct_ values of [Ru_*x*_|Co_*y*_]_3_[Fe]_6_ and the *E*_pa_ vs the different amounts (%) of complexes **1** and **2**. The measurements were performed in ACN, 0.1 M TBAPF_6_ with ITO/PET, Pt wire, and Ag/Ag^+^ wire as the working, counter, and reference electrodes, respectively.

The electrochemical differences between the regions A, B, and C is shown Figure 2C for [Ru_*x*_|Co_*y*_]_*n*_[Fe]_6_. In region A, no electrochemical oxidation of complex **3** is visible. The low percentage of the ruthenium complex **1** (blue) does not mediate electron transfer. In region B (red) and C (black), the percentage of complex **1** is sufficiently high to allow electron transfer within the assembly. The oxidation potentials are significantly shifted to 1.5 V (region B) and 1.3 V (region C) in comparison to that of [Fe]_6_ (1.1 V) (Figure S1). This overpotential is a result of the presence of the insulating cobalt complexes **2**. Additional discussion about regions A and C are included in the Supporting Information, as well as additional data of region B (Figures S7–S9). The films were analyzed using electrochemical impedance spectroscopy (EIS) to obtain information regarding their charge transfer resistance (*R*_ct_) (Figure 3A). We observed that the *R*_ct_ value becomes smaller upon increasing the amount of the ruthenium complexes (**1**). For region A, the amount of complex **1** is low, resulting in high *R*_ct_ values (230–50 kΩ) and no electrochromic activity. For regions B and C, since complex **1** is more dominant within [Ru_*x*_|Co_*y*_]_*n*_, a lower voltage is required to oxidize the iron (**3**) layer (Figure 3B); the charge remains trapped in this layer. The resistance of these films is also lower (*R*_ct_ = 20–0.5 kΩ). For example, [Ru_40_|Co_60_]_3_[Fe]_6_ shows higher resistance toward charge transfer (*R*_ct_ ≈ 20 kΩ) than [Ru_90_|Co_10_]_4_[Fe]_6_ (*R*_ct_ ≈ 0.5 kΩ) (Figures 3A and S10). The EIS measurements confirm that the ruthenium complexes (**1**) lower the resistance toward the oxidation of iron complexes (**3**), whereas the cobalt complexes (**2**) insulate components with respect to the WE surface. The EIS results confirm the observation we achieved using voltammetry methods.

**Figure 4:**
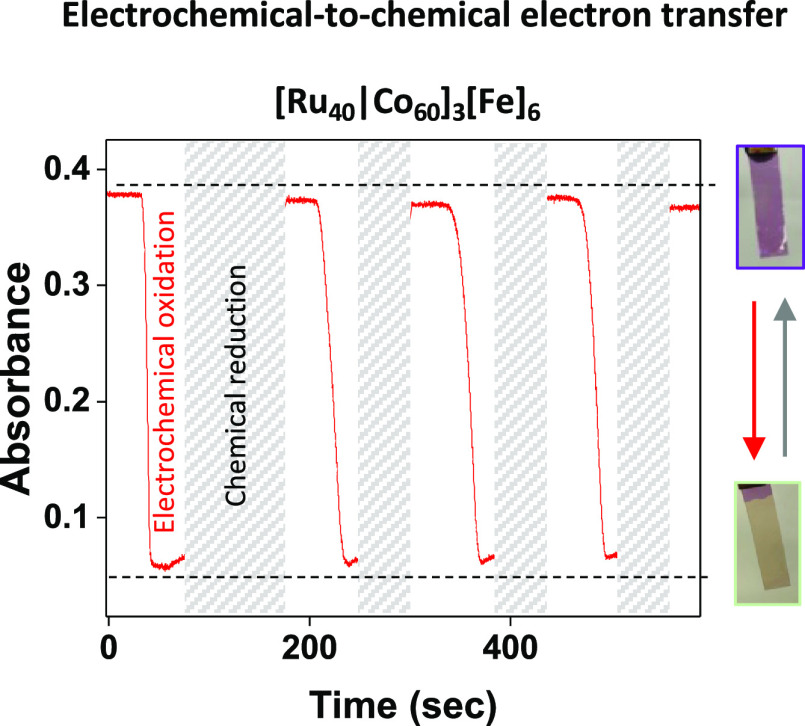
UV/vis measurements at λ = 570 nm of consecutive electrochemical oxidations (red) and chemical reductions (gray) of [Ru_40_|Co_60_]_3_[Fe]_6_. This film was dipped for 2 s in an aqueous solution of [Fe(CN)_6_]^4–^ (5.0 mM). The charge release is indicated by the instant reappearance of the purple color (right, photos). Subsequently, the film was washed with water before the absorbance measurement.

The trapped charge can be released upon demand by exposing the oxidized films to a chemical reduction agent, resulting in electrochemical-to-chemical energy transfer. The orange films turned purple within 2 s in an aqueous solution containing ferrocyanide (5 mM), owing to the low redox potential of the ferrocyanide (*E*_ox_ = 0.35 V) (Scheme 1D). This observation suggests an outer-sphere electron transfer between the ferrocyanide and the on-surface trapped [Fe^3+^] of the redox-active layer. The reversibility of this redox process was evaluated using a combination of spectroelectrochemical measurements (for charge trapping) and the abovementioned charge release procedure. Upon applying 1.6 V, the MLCT band of the divalent iron complexes **3** (λ_max_ = 570 nm) was reduced from 0.37 to 0.05, as one would expect for the formation of Fe^3+^ species (Figure 4). This characteristic band reappears upon chemical reduction. This charge trapping–release procedure was repeated eight times, indicating a stable and reversible electrochemical/chemical redox pathway. A control measurement in distilled water showed that the chemical reduction reagent is essential to release the charge (Figure S11).

**Figure 5:**
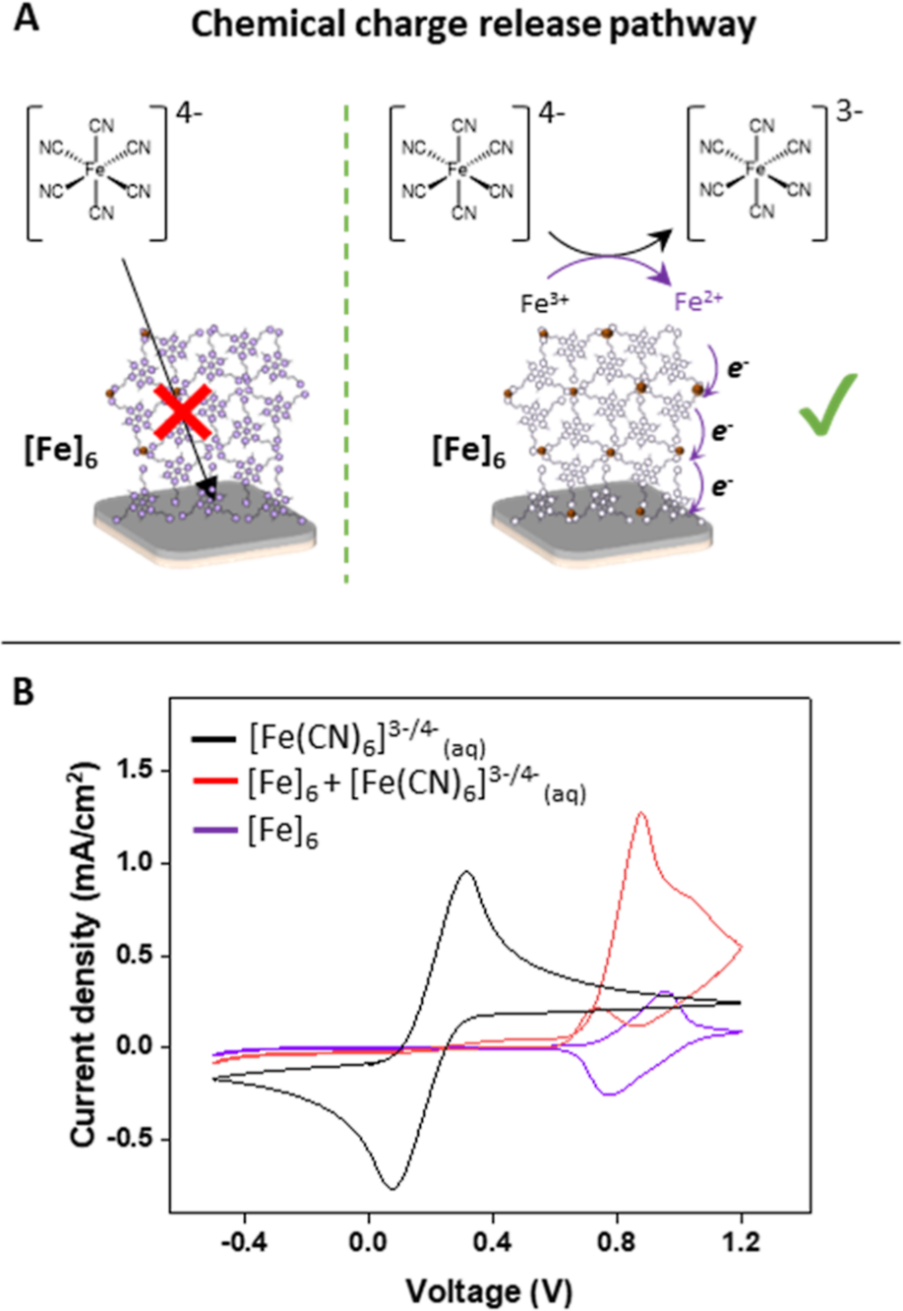
(A) Suggested reaction pathways. (B) CV curves in aqueous ferrocyanide solutions (5.0 mM) using bare ITO/PET (black) and [Fe]_6_ on ITO/PET (red). The purple curve is the CV curve of [Fe]_6_ on ITO/PET without ferrocyanide in solution. The measurements were performed in aqueous 0.1 M KCl solutions with Pt wire and Ag/Ag^+^ wire as counter and reference electrodes, respectively (scan rate = 100 mV/s).

The mechanism underlying the chemical charge release is not trivial: where does the electron transfer take place? Two scenarios are plausible: (i) the reduction agent diffuses into the film structure, followed by electron transfer, and (ii) the electron transfer takes places at the thin film–water interface (Figure 5A).

The latter process includes a cascade of electron transfer processes between the iron complexes of the MAs. To distinguish between these two possibilities, we carried out the following experiments using six layers of the iron complex (**3**) to model the top layer [Fe]_6_. The CV curve of this [Fe]_6_ layer on ITO/PET shows a reversible redox process; no charge trapping occurs (Figures S1 and 5B, purple). The CV curve of bare ITO/PET in an aqueous solution of ferrocyanide (5.0 mM) showed the oxidation and reduction of this chemical agent (range: −1.0 to 1.1 V) (Figure 5B, black). However, no electrochemical signal of the ferrocyanide was observed at the expected voltage (*E*^0^ = 0.2 V) when the working electrode was modified with [Fe]_6_. This observation indicates that the modified electrode prohibits the ferrocyanide from interacting directly with the metal oxide surface of the working electrode. By scanning to higher potentials, we observed an oxidative catalytic peak starting at the onset potential (*V*_ox_ = 0.7 V) of complex **3** (Figure 5B, red) followed by another peak related to the oxidation of the film. The first oxidation wave suggests a continuous regeneration of the film by ferrocyanide. The relatively small electrochemical reduction peak (from the [Fe]_6_) confirms the electrochemical and chemical isolation of the chemical agent in solution. Although other pathways cannot be rigorously excluded, these results make an electron transfer process at the thin film–water interface likely.

## Conclusions

The inclusion of cobalt complexes in layers of ruthenium complexes can be used to control the electron transfer pathways between the metal oxide surfaces and the nanoscale layers of isostructural iron complexes. These iron complexes are positioned >70 nm from the electrode surfaces and can be fully insulated or electrochemically addressed, dependent on the ratio between the cobalt and ruthenium complexes. The colorless cobalt complex is electrochemically inactive to ITO; however, it shows one-electron oxidation of Co^2+/3+^ mediated by the ruthenium complex.^59,60^ Therefore, a relatively high amount of cobalt complexes blocks the electron transfer between the top iron layer and the ITO. Increasing the amount of the ruthenium complexes allows electron transfer to occur, which can also involve the cobalt complexes. This electrochemical behavior is reflected in the color changes. We can trap positive charges mainly in the top layer consisting of the iron complexes. Interestingly, the potential of these assemblies for energy storage and conversion was conceptually demonstrated by the efficient release of the electrochemically stored charge in the layers of the iron complexes. The release of the trapped charges in our system occurs at the film–solution interfaces rather than diffusion of the electron donor into the film.

The solution-to-surface assembly of the mixtures of cobalt and ruthenium complexes results in films with compositions that do not reflect the molecular ratios in solution. The amount of ruthenium complex **1** is lower in the assembly than in solution; simultaneously, the amount of cobalt complex **2** is higher in the assembly. This observation shows that the formation of such films is not trivial. However, the combination of just two layers of three isostructural metal complexes in different ratios resulted in a series of conceptually new functional materials.

## Supporting Information Available

Experimental details, materials and methods, discussion about the electrochemical properties of regions A and C, and spectroscopic and electrochemical characterization of the films (PDF)

## Abbreviations

MA: molecular assembly
CV: cyclic voltammetry
[Ru_*x*_|Co_*y*_]_*n*_: *x* and *y* represent the percentage of the ruthenium (**1**) and cobalt (**2**) complexes in the feeding solutions, respectively, and *n* is the number of depositing cycles
