# Supplementary material for: Compositionally Controlled Electron Transfer in Metallo-Organics
Source: J Am Chem Soc. 2023 Aug 2;145(32):18075–83. doi: 10.1021/jacs.3c05874 (PMC10436274; doi:10.1021/jacs.3c05874)
Supplement: Supplementary file 1 — ja3c05874_si_001.pdf [file ja3c05874_si_001.pdf]

SUPPORTING INFORMATION

Compositionally Controlled Electron Transfer in Metallo-Organic Assemblies

Yonatan Hamo[1], Alena Neudert[1], Tatyana Bendikov[2],
Michal Lahav[1,*], and Milko E. van der Boom[1,*]

[1]Department of Molecular Chemistry and Materials Science, [2]Department of Chemical Research Support. The Weizmann Institute of Science, 7610001 Rehovot, Israel. Email: michal.lahav@weizmann.ac.il, milko.vanderboom@weizmann.ac.il

Experimental section

Materials and Methods

Solvents (AR-grade) were purchased from Bio-Lab (Jerusalem), Frutarom (Haifa, Israel), or Mallinckrodt Baker (Phillipsburg, NJ). $PdCl_2(PhCN)_2$, tetrabutylammonium hexafluorophosphate ($TBAPF_6$), and anhydrous ACN (> 99.9%) were purchased from Sigma-Aldrich. Complexes **1-3** were prepared as reported.[S1-S3] Indium-tin oxide (ITO)-coated poly(ethylene terephthalate) (PET) substrates (10 cm × 10 cm, Rs = 30 Ω/sq) were purchased from Xinyan Technology, Ltd. (Hong Kong, China). The ITO/PET substrates were cleaned with acetone, then with a UVOCS cleaning system (Montgomery, PA) for 6 min, and washed with ethanol. A Laurell spin-coater, model WS-400A-6NPP/LITE, was used for fabricating the molecular assemblies (MAs).

UV/Vis Spectroscopy. UV/Vis spectra were recorded on a Cary 100 spectrophotometer. The absorbance was measured using the Cary Win UV–Scan application program, version 3.00 (182) by Varian (350–800 nm). The transmittance was measured using the Cary WinUV–Kinetics application program, version 3.00 (182) by Varian. Bare substrates were used to compensate for the background absorption.

Electrochemical Characterization. Electrochemical experiments were carried out using a CHI760E electrochemical workstation. The following configuration of the electrochemical cell was used: ITO/PET (0.5 cm × 1.5 cm) served as the working electrode, Ag/Ag^+ was used as the reference electrode, and a Pt wire was used as the counter electrode. Tetrabutylammonium hexafluorophosphate ($TBAPF_6$) in ACN (0.1 M) was used as the supporting electrolyte. Spectroelectrochemistry measurements were performed in a N_2-filled glovebox for Region C.

Focused Ion Beam (FIB) Microscopy. The lamella was prepared using a dual beam FIB-SEM Helios 600. The images were taken at cross-sections that were generated by milling the sample with a 30 keV Ga^+ FIB. The sample was first locally coated with a 150–200 nm-thick layer of platinum using electron beam-assisted deposition, which was followed by the ion beam-assisted deposition of a 500–600 nm-thick layer of platinum. This coating protects the molecular assembly from ion-beam damage, providing a clean edge of the cross-section. High angle annular dark field (HAADF) STEM imaging and EDS measurements were carried out using a Thermo Fisher Scientific Themis Z TEM, which is double aberration-corrected and equipped

with a Super-X large solid angle X-ray detector for EDS. The measurements were carried out using 200 kV acceleration voltage.

X-ray photoelectron spectroscopy (XPS). The measurements were carried out with a Kratos AXIS ULTRA system using a monochromatic Al Kα X-ray source (hν = 1486.6 eV) at 75 W and detection pass energies ranging between 20 and 80 eV. Curve fitting analysis was based on linear or Shirley background subtraction and application of Gaussian-Lorenzian line shapes.

Fabrication of the Molecular Assemblies (MAs). The metallo-organic assemblies were obtained by iterative spin-coating of solutions of $PdCl_2(PhCN)_2$ (THF, 3.6 mM), a solution containing complexes **1** and **2** (0-100% of **1** in steps of 10% in CH_2Cl_2:MeOH = 1:1 v/v; 0.6 mM total) or a solution of complex **3** (CH_2Cl_2:MeOH = 1:1 v/v; 0.6 mM). The $PdCl_2(PhCN)_2$ solution (0.5 mL) was drop-casted onto ITO/PET (3 cm × 3 cm), the substrate was spun at 500 rpm for 10 s, and then at 1000 rpm for 30 s. Next, a solution containing complexes **1** and **2**, or complex **3** was drop-casted (0.5 mL) and the substrates were spun as described above. The substrates were washed with acetone. The deposition of $PdCl_2(PhCN)_2$ and a mixture of **1** and **2,** or only **3** are referred to as a single deposition cycle. For the formation of the MAs, n = 3-4 deposition cycles were carried out with mixtures of **1** and **2** and then, six deposition cycles using complex **3**.

Discussion about the electrochemical properties of Regions A [Ru < 30%] and C [Ru > 70%]. The electrochemical behavior of $[Ru_x|Co_y]_3$ in Region B [40% < Ru < 60%] was discussed in the main text and shown **Figure S8**. Here we focus on $[Ru_{20}|Co_{80}]_3$, $[Ru_{20}|Co_{80}]_3[Fe]_6$ and $[Ru_{90}|Co_{10}]_4$, $[Ru_{90}|Co_{10}]_4[Fe]_6$ as representative examples of Region A (**Figure S7**) and Region C (**Figure S9**), respectively.

Region A [Ru < 30%]. For $[Ru_{20}|Co_{80}]_3$, low anodic and cathodic currents (i_p = 0.02 A cm^{-2}, $Q_{1st\ cycle}$ = 0.038 mC cm^{-2} at V_{ox} = 1.25 V and V_{red} = 1.15 V) were observed. The observed redox activity corresponds to the oxidation and reduction of ruthenium complexes **1** that are directly attached or in proximity to the electrode (**Figure S7B**). The excess of electrochemically silent complex (**2**) hampers the electron-transfer between the ruthenium complex (**1**) and the electrode surface, as indicated by charge trapping ($Q_{2st\ cycle}$ = 0.012 mC cm^{-2}).

After adding a layer of iron complex **3** (six deposition cycles), the following results were observed. For $[Ru_{20}|Co_{80}]_3[Fe]_6$, we did not observe redox activity for the

top layer consisting of iron complexes (**3**), since $[Ru_{20}|Co_{80}]_3$ acts as an insulator with respect to complex **3** (**Figure S7B**). The amount of redox-active ruthenium complexes (**1**) is clearly not sufficient to allow efficient electron transport from the top layer (**3**). The distance between the ITO surface and the iron complexes (**3**) is too large for direct communication (~78 nm by FIB-SEM).

Region C [Ru > 70%]: Increasing the amount of complex **1** with a concurrent decrease of complex **2** in $[Ru_x|Co_y]_4$ derived from a feeding solution containing 70-100% complex **1** led to less significant differences in the oxidative charges involved in the 1th and 2nd CVs ($Q_{1st\ cycle}$ = 0.48 mC cm^{-2}, $Q_{2nd\ cycle}$ = 0.46 mC cm^{-2} for $[Ru_{90}|Co_{10}]_4$) (**Figure S9C**). Note that as the relative amount of complex **1** is higher, the thickness of the film is lower for the same number of deposition steps, allowing direct communication between iron complex **3** and the electrode surface (**Figure S2**). This effect is manifested by the additional reduction peak at ~1.0 V. Therefore an additional deposition step of complexes **1** and **2** was applied. There is a neglectable contribution of the cobalt complex (**2**) to the oxidative current (**Figure S9C**). Thus, the CVs resemble the electrochemical data observed for a layer consisting exclusively of ruthenium complex **1**. Upon oxidation of complex **1** (V_{ox} = 1.25 V), the thin film becomes transparent and regains its initial orange color during the reduction process (V_{red} = 1.15 V). Continued cycling resulted in repetitive CV curves resembling the second cycles, each by current, shape, and redox potential for regions B and C.

After adding a layer of iron complex **3** (six deposition cycles), the following results were observed. For $[Ru_{90}|Co_{10}]_4[Fe]_6$, CV measurement from 0.2 V to 1.8 V showed two peaks at 1.2 V and 1.4 V, associated with the oxidation of both the ruthenium (**1**) and iron complexes (**3**). For the reduction, only one peak was observed, corresponding to the formation of divalent **1** ($Ru^{3+ \rightarrow 2+}$), whereas positive charges remained trapped as Fe^{3+} in the top layer (**Figure S9C'**). The area beneath the first oxidation peak ($Q_{1st\ cycle}$ = 2.20 mC cm^{-2}) corresponds to the oxidation of both complexes **1** and **3**, whereas the area beneath additional cycles corresponds to complex **1** only, $Q_{2nd\ cycle}$ = 0.85 mC cm^{-2}. Subtracting the second cycle from the first cycle results in a ratio of complex **1**:**3** = 1:1.3 which is in agreement with the number of deposited cycles.

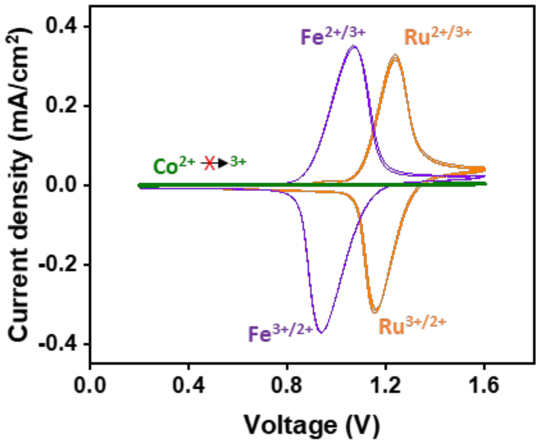

Figure S1. Cyclic voltammetry of $[Co]_4$ (green), $[Fe]_6$ (purple), and $[Ru]_6$ (orange). ITO/PET, scan rate = 0.1 V/s, 0.1 M $TBAPF_6$ in ACN. Pt wire and Ag wire were used as the counter and reference electrodes, respectively.

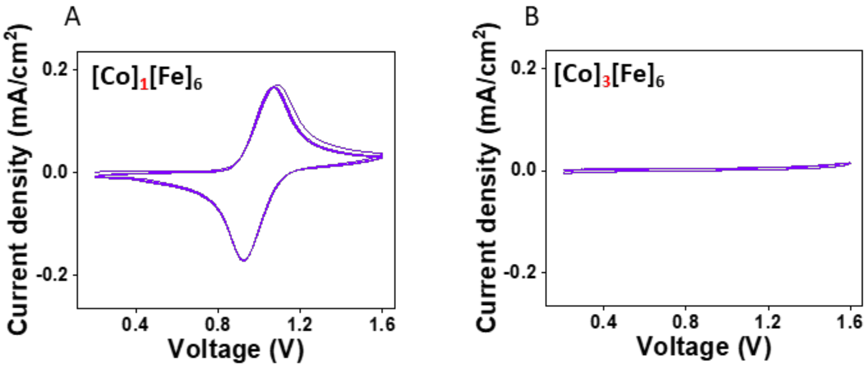

Figure S2. The number of $[Co]_x$ deposition cycles (x = 1 and 3) needed to insulate the top $[Fe]_6$ layer from [ITO/PET]. Cyclic voltammograms of A) $[Co]_1[Fe]_6$ and B) $[Co]_3[Fe]_6$. ITO/PET, scan rate = 0.1 V/s, 0.1 M $TBAPF_6$ in ACN. Pt wire and Ag wire were used as the counter and reference electrodes, respectively.

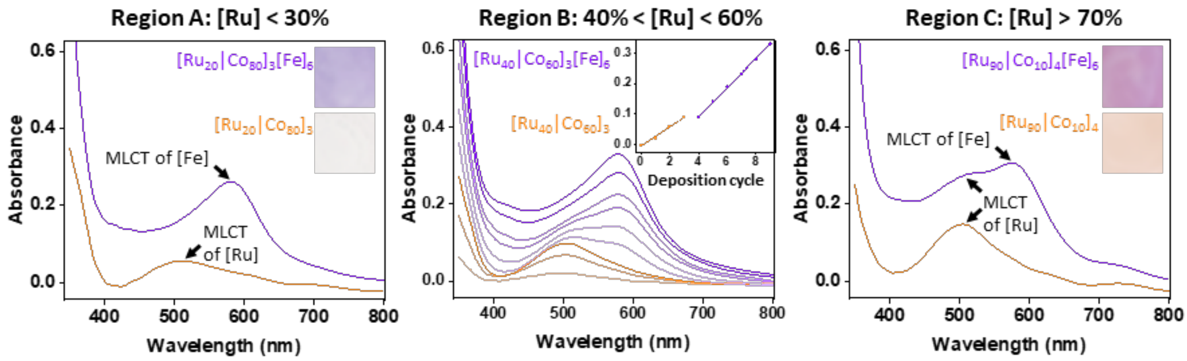

Figure S3. Formation of the molecular assemblies on ITO/ PET. Left, region A: UV/Vis spectra of $[Ru_{20}|Co_{80}]_3$ (orange) and $[Ru_{20}|Co_{80}]_3[Fe]_6$ (purple). Center: region B: UV/Vis spectra recorded after each deposition cycle: $PdCl_2(PhCN)_2$, a mixture of ruthenium (**1**) and cobalt (**2**) complexes (orange) and iron complex **3** (purple). Inset: Linear fits of the absorbance intensities of the MLCT bands of **1** (λ_{max} = 500 nm) and **3** (λ_{max} = 570 nm) *vs* the number of deposition cycles. For both fits, $R^2 > 0.99$. Right, UV/Vis spectra of region C: $[Ru_{90}|Co_{10}]_4$ (orange) and $[Ru_{90}|Co_{10}]_4[Fe]_6$ (purple). The insets of regions A and C show the photographs of the surfaces of the films (1.5 cm × 1.5 cm).

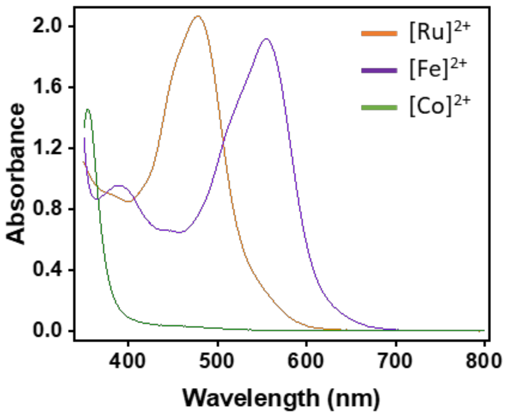

Figure S4. UV/Vis spectra of complexes **1** (Ru^{2+}, orange), **2** (Co^{2+}, green), and **3** (Fe^{2+},purple) in solution (0.6 mM in MeOH:DCM = 1:1 v/v).

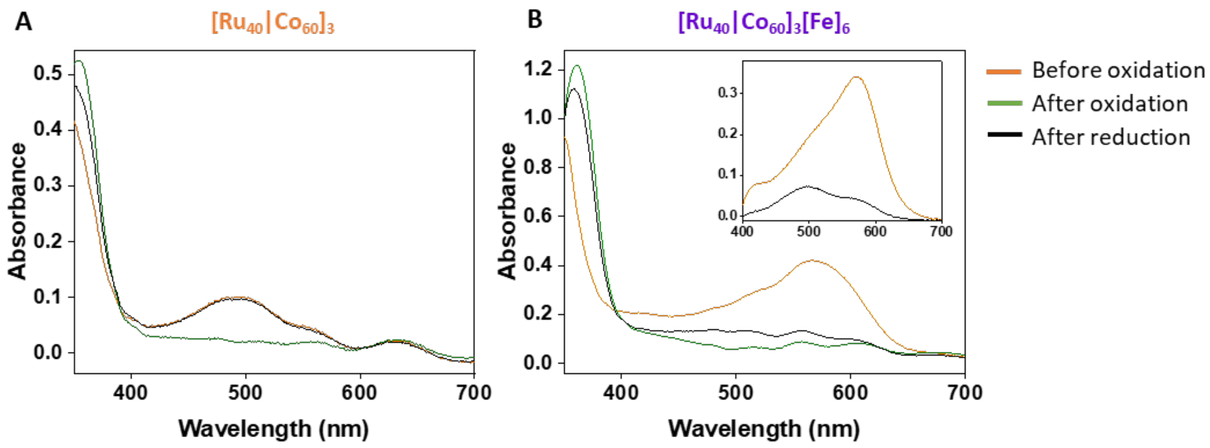

Figure S5. A) Spectroelectrochemistry of $[Ru_{40}|Co_{60}]_3$ on ITO/PET. The UV/Vis spectra of the molecular assemblies were recorded before (orange) and after (green) applying an oxidative potential step of V_{ox} = 1.6 V for 60 s. The assemblies were reduced by applying a potential step of V_{red} = 0.2 V (10 s; black). B) Spectroelectrochemical data of $[Ru_{40}|Co_{60}]_3[Fe]_6$ on ITO/PET. The UV/Vis spectra were recorded before (orange) and after (green) applying an oxidative potential step of V_{ox} = 1.6 V for 60 s. $[Ru_{40}|Co_{60}]_3[Fe]_6$ was reduced by applying a potential step of V_{red} = 0.2 V (10 s; black). Inset: UV/Vis spectra after subtraction of the green spectra (oxidized state) from the ground state (orange) and from the reduced state (black). The measurements were performed in a 0.1 M acetonitrile solution of $TBAPF_6$ with ITO/PET, Pt wire, and Ag/Ag^+ wire as the working, counter and reference electrodes, respectively.

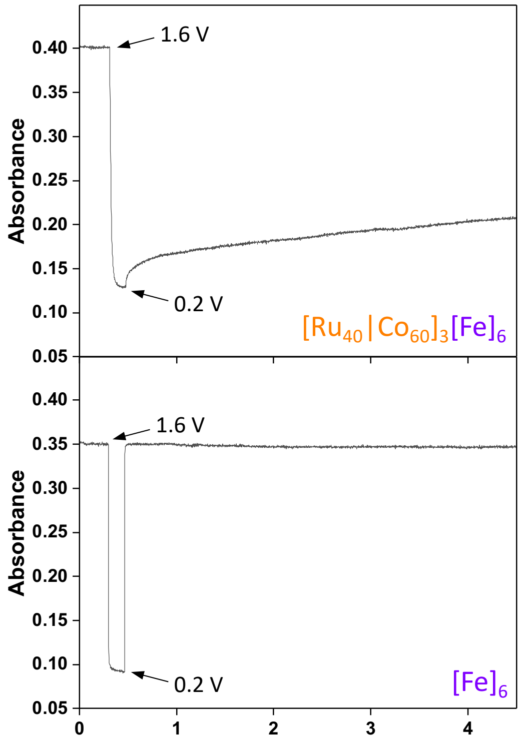

Figure S6. Spectroelectrochemical measurements (λ = 570 nm). Top: $[Ru_{40}|Co_{60}]_3[Fe]_6$ 1.6 V, 10 s; 0.2 V, 10 s, open circuit potential. Bottom: $[Fe]_6$ 1.6 V, 10 s; 0.2 V, 10 s, open circuit potential. The measurements were performed in a 0.1 M ACN solution of $TBAPF_6$ with ITO/PET, Pt wire, and Ag/Ag^+ wire as the working, counter and reference electrodes, respectively.

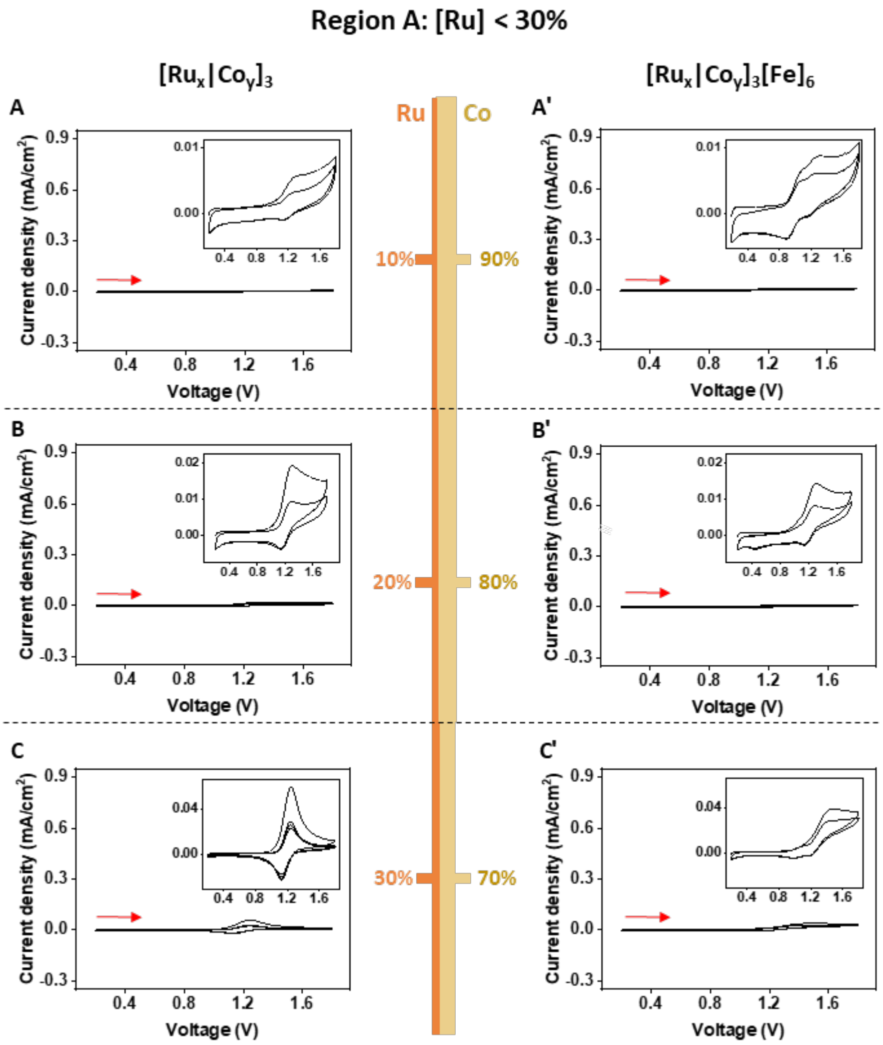

Figure S7. CV measurements of region A. A) $[Ru_{10}|Co_{90}]_3$, B) $[Ru_{20}|Co_{80}]_3$, C) $[Ru_{30}|Co_{70}]_3$, A') $[Ru_{10}|Co_{90}]_3[Fe]_6$, B') $[Ru_{20}|Co_{80}]_3[Fe]_6$, and C') $[Ru_{30}|Co_{70}]_3[Fe]_6$. The red arrows show the scan direction. The insets are enlarged main graphs. Scan rate = 0.1 V/s, 0.1 M $TBAPF_6$ in ACN with ITO/PET, Pt wire, and Ag/Ag^+ wire as the working, counter and reference electrodes, respectively.

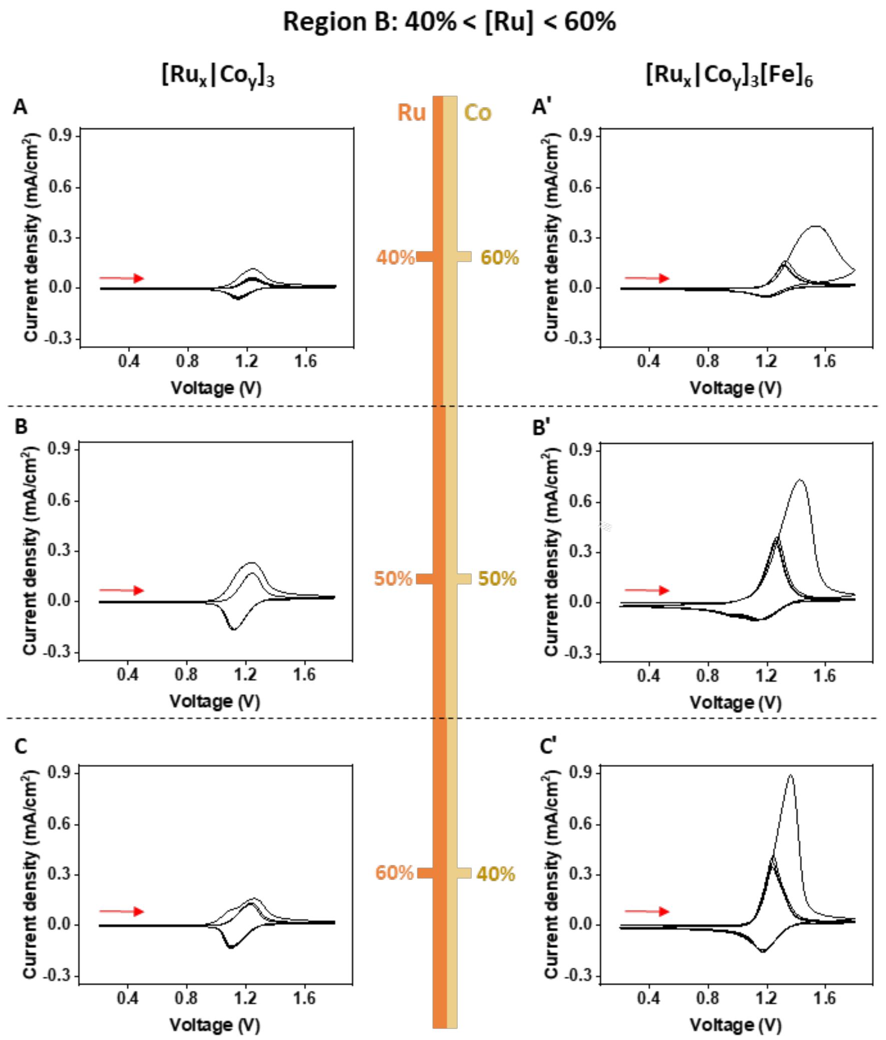

Figure S8. CV measurements of region B. A) $[Ru_{40}|Co_{60}]_3$, B) $[Ru_{50}|Co_{50}]_3$, C) $[Ru_{60}|Co_{40}]_3$, A') $[Ru_{40}|Co_{60}]_3[Fe]_6$, B') $[Ru_{50}|Co_{50}]_3[Fe]_6$, and C') $[Ru_{60}|Co_{40}]_3[Fe]_6$. The red arrows show the scan direction. Scan rate = 0.1 V/s, 0.1 M $TBAPF_6$ in ACN with ITO/PET, Pt wire, and Ag/Ag^+ wire as the working, counter and reference electrodes, respectively.

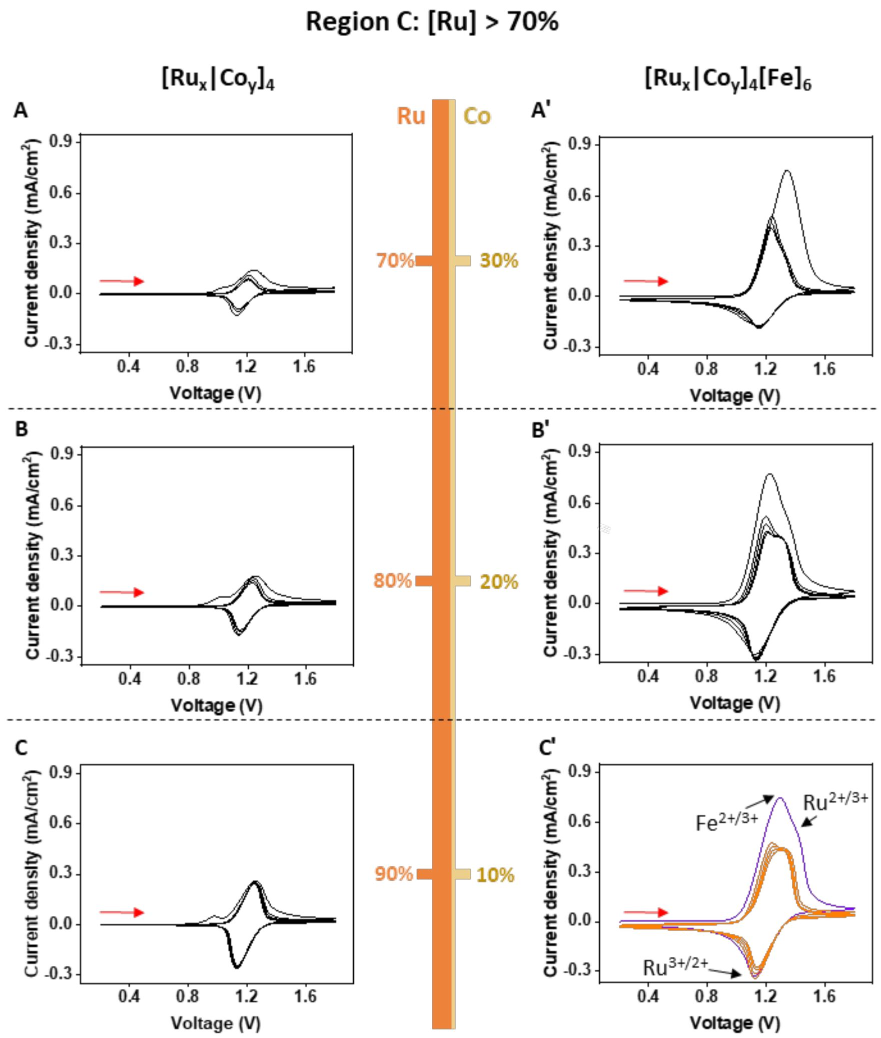

Figure S9. CV measurements of region C. A) [Ru$_{70}$|Co$_{30}$]$_4$, B) [Ru$_{80}$|Co$_{20}$]$_4$, C) [Ru$_{90}$|Co$_{10}$]$_4$, A') [Ru$_{70}$|Co$_{30}$]$_4$[Fe]$_6$, B') [Ru$_{80}$|Co$_{20}$]$_4$[Fe]$_6$, and C') [Ru$_{90}$|Co$_{10}$]$_4$[Fe]$_6$. The red arrows show the scan direction. Scan rate = 0.1 V/s, 0.1 M TBAPF$_6$ in with ITO/PET, Pt wire, and Ag/Ag$^+$ wire as the working, counter and reference electrodes, respectively.

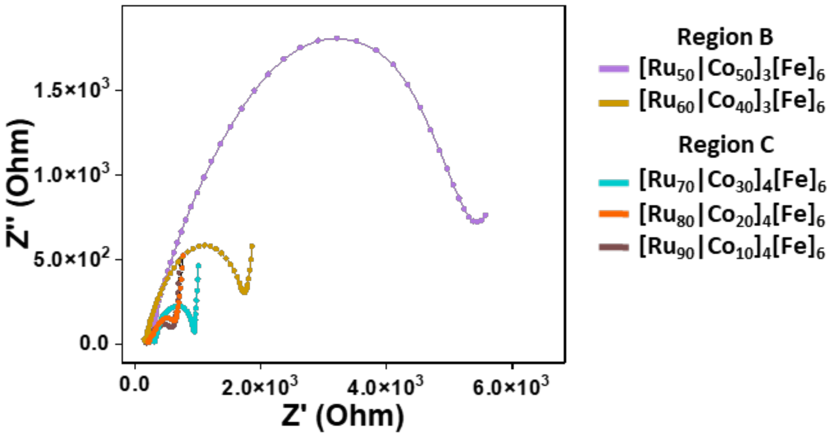

Figure S10. Nyquist plots of $[Ru_x|Co_y]_{3\text{-}4}[Fe]_6$ derived from the electrochemical impedance spectroscopy (EIS) in ACN, 0.1 M $TBAPF_6$, with ITO/PET, Pt wire, and Ag/Ag^+ wire as the working, counter and reference electrodes, respectively. (Frequencies: 0.1 – 10^6 Hz, Amplitude: 10 mV, E^o = 1.2 V).

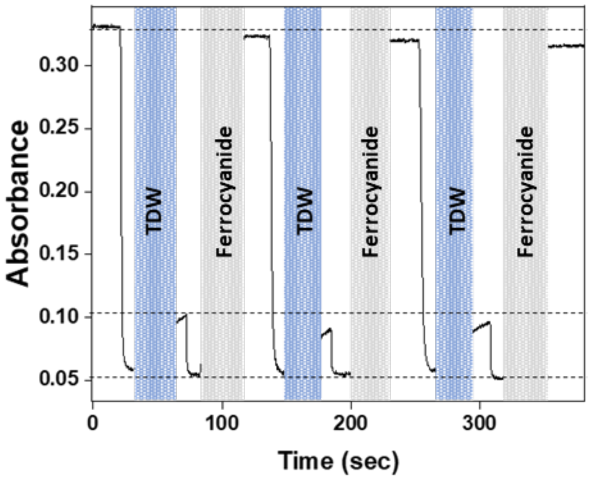

Figure S11. Control measurements of the chemical charge release. UV/Vis measurements at λ = 570 nm of the electrochemical oxidation, the film was oxidized upon 1.8 V, 10 s. The blue area represents the dipping of the oxidized $[Ru_{40}|Co_{60}]_3[Fe]_6$ in water for 2 s. Subsequently, the film was washed with water before the absorbance measurement. The grey area represents the immersion of the oxidized $[Ru_{40}|Co_{60}]_3[Fe]_6$ in 5.0 mM ferrocyanide aqueous solution for 2 s followed by washing with triple distilled water (TDW).

References

(S1) Malik, N.; Elool Dov, N.; de Ruiter, G.; Lahav, M.; van der Boom, M. E. On-Surface Self-Assembly of Stimuli-Responsive Metallo-Organic Films: Automated Ultrasonic Spray-Coating and Electrochromic Devices *ACS Appl. Mater. Interfaces* **2019**, *11*, 22858–22868.

(S2) Choudhury, J.; Kaminker, R.; Motiei, L.; de Ruiter, G.; Morozov, M.; Lupo, F.; Gulino, A.; van der Boom, M. E. Linear vs Exponential Formation of Molecular-Based Assemblies *J. Am. Chem. Soc.* **2010**, *132*, 9295–9297.

(S3) Shankar, S.; Lahav, M.; van der Boom, M. E. Coordination-based Molecular Assemblies as Electrochromic Materials: Ultra-high Switching Stability and Coloration Efficiencies. *J. Am. Chem. Soc.* **2015**, *137*, 4050–4053.
